# Are Older Adults Planning for their Transportation Futures? A National Survey on Driving Retirement

**DOI:** 10.1093/geroni/igaf122.2011

**Published:** 2025-12-31

**Authors:** Alycia Bayne, Alexa Siegfried, Lindsey Witt-Swanson

**Affiliations:** National Opinion Research Center at The University of Chicago, Chicago, Illinois, United States; National Opinion Research Center at The University of Chicago, Chicago, Illinois, United States; National Opinion Research Center at The University of Chicago, Chicago, Illinois, United States

## Abstract

Driving a personal vehicle is the preferred mode of transportation among older adults. However, many older adults will outlive their ability to drive due to age-related changes in vision, cognition, and physical function. Despite the impact of transportation on health, wellbeing, independence, and mobility, few older adults plan for their driving retirement (i.e., the time when they permanently stop driving). Research is needed to understand how older adults plan for their driving retirement, including who they trust to talk with them about this transition. To explore this topic, NORC conducted a nationally representative survey of 1,010 U.S. adults aged 50+ using the NORC Foresight 50+ Panel (May 2024), which is drawn from the NORC national sample frame that has over 97% coverage of U.S. addresses. We found that older adults trust a spouse or partner (51%), adult child (48%), or primary care provider (41%) to have conversations with them about driving retirement. Few older adults (31%) have a plan in place for how they will travel when they can no longer drive. Adults aged 75+ are more worried about not being able to drive in the future, compared to adults aged 50 to 64. Demographic factors affect driving retirement planning. This study provides new data that can be used by public health, transportation practitioners, caregivers, and others to support older adults in proactively planning for their transportation futures.

